# Cost-Effectiveness of Antivenoms for Snakebite Envenoming in 16 Countries in West Africa

**DOI:** 10.1371/journal.pntd.0004568

**Published:** 2016-03-30

**Authors:** Muhammad Hamza, Maryam A. Idris, Musa B. Maiyaki, Mohammed Lamorde, Jean-Philippe Chippaux, David A. Warrell, Andreas Kuznik, Abdulrazaq G. Habib

**Affiliations:** 1 College of Health of Sciences, Bayero University, Kano, Nigeria; 2 Infectious Diseases Institute, Makerere University College of Health Sciences, Kampala, Uganda; 3 Institut de Recherche pour le Development, Cotonou, Benin Republic and Université Paris Descartes, Sorbonne Paris Cité, Faculté de Pharmacie, Paris, France; 4 Nuffield Department of Medicine, University of Oxford, Oxford, United Kingdom; 5 Celgene Corporation, Warren, New Jersey, United States of America; University of Kelaniya, SRI LANKA

## Abstract

**Background:**

Snakebite poisoning is a significant medical problem in agricultural societies in Sub Saharan Africa. Antivenom (AV) is the standard treatment, and we assessed the cost-effectiveness of making it available in 16 countries in West Africa.

**Methods:**

We determined the cost-effectiveness of AV based on a decision-tree model from a public payer perspective. Specific AVs included in the model were Antivipmyn, FAV Afrique, EchiTab-G and EchiTab-Plus. We derived inputs from the literature which included: type of snakes causing bites (carpet viper (*Echis* species)/non-carpet viper), AV effectiveness against death, mortality without AV, probability of Early Adverse Reactions (EAR), likelihood of death from EAR, average age at envenomation in years, anticipated remaining life span and likelihood of amputation. Costs incurred by the victims include: costs of confirming and evaluating envenomation, AV acquisition, routine care, AV transportation logistics, hospital admission and related transportation costs, management of AV EAR compared to the alternative of free snakebite care with ineffective or no AV. Incremental Cost Effectiveness Ratios (ICERs) were assessed as the cost per death averted and the cost per Disability-Adjusted-Life-Years (DALY) averted. Probabilistic Sensitivity Analyses (PSA) using Monte Carlo simulations were used to obtain 95% Confidence Intervals of ICERs.

**Results:**

The cost/death averted for the 16 countries of interest ranged from $1,997 in Guinea Bissau to $6,205 for Liberia and Sierra Leone. The cost/DALY averted ranged from $83 (95% Confidence Interval: $36-$240) for Benin Republic to $281 ($159–457) for Sierra-Leone. In all cases, the base-case cost/DALY averted estimate fell below the commonly accepted threshold of one time per capita GDP, suggesting that AV is highly cost-effective for the treatment of snakebite in all 16 WA countries. The findings were consistent even with variations of inputs in 1—way sensitivity analyses. In addition, the PSA showed that in the majority of iterations ranging from 97.3% in Liberia to 100% in Cameroun, Guinea Bissau, Mali, Nigeria and Senegal, our model results yielded an ICER that fell below the threshold of one time per capita GDP, thus, indicating a high degree of confidence in our results.

**Conclusions:**

Therapy for SBE with AV in countries of WA is highly cost-effective at commonly accepted thresholds. Broadening access to effective AVs in rural communities in West Africa is a priority.

## Author Summary

Antivenom is the main intervention against snakebite poisoning but is relatively scarce, unaffordable and the situation has been compounded further by the recent cessation of production of effective antivenoms and marketing of inappropriate products. Given this crisis, we assessed the cost effectiveness of providing antivenoms in West Africa by comparing costs associated with antivenom treatment against their health benefits in decreasing mortality. In the most comprehensive analyses ever conducted, it was observed the incremental cost effectiveness ratio of providing antivenom ranged from $1,997 in Guinea Bissau to $6,205 for Liberia and Sierra-Leone per death averted while cost per Disability Adjusted Life Year (DALY) averted ranged from $83 for Benin Republic to $281 for Sierra-Leone. There is probability of 97.3–100% that antivenoms are very cost-effective in the analyses. These demonstrate antivenom is highly cost-effective and compares favorably to other commonly funded healthcare interventions. Providing and broadening antivenom access throughout areas at risk in rural West Africa should be prioritized given the considerable reduction in deaths and DALYs that could be derived at a relatively small cost.

## Introduction

Snakebite poisoning is a significant cause of death and disability in rural West Africa [1,2,3,4,5,6,7]. The exact burden of snakebite is difficult to ascertain and is often undereported. A study by Jean-Philippe Chippaux reported an estimate of over 314, 000 envenomations, 7300 mortality and nearly 6000 amputations occurring yearly in sub-Saharan Africa (SSA) [7]. However, even in West Africa alone, a range of 1504 to 18,654 annual mortality from snakebite envenoming has been made [8]. This is further compounded by the variability in snakebite incidence with estimates of as high as 500 bites per 100,000 persons per year in parts of northern Nigeria [9].

Vipers (*Echis ocellatus*, *E*. *leucogaster* and *E*. *jogeri)* are a major cause of snakebite envenoming throughout the sub-region mainly in Benin republic, Burkina Faso, Cameroun, Chad, Gambia, Ghana, Mali, Niger, Nigeria, Togo and Senegal [1,2,3,4,5,6,7]. In the sub-region, envenoming from snakes other than vipers mostly results from African spitting cobras (*Naja nigricollis*, *N*. *katiensis*), puff-adder (*Bitis arietans*), mambas (*Dendroaspis viridis*, *D*. *polylepis*), burrowing asps or stiletto snakes (*Atractaspis* species), night adders (*Causus maculatus*, *C*. *rhombeatus*, *C*. *resimus*, *C*. *lichtensteinii*) and very rarely boomslang (*Dispholidus typus*). Joger’s carpet viper (*E*. *jogeri*) is confined to Mali. Romane’s carpet viper (*Echis leucogaster*) and Egyptian cobras (*Naja haje* and *N*. *senegalensis*) are causes of snakebite envenoming in the Sahelian and drier parts of West Africa while the forest cobra (*Naja melanoleuca*) and the Gaboon viper (*Bitis gabonica*) cause occasional bites in the rain forest and South-eastern parts of the sub-region [1,5,7].

In West Africa, carpet vipers may account for as many as two thirds of all snakebite envenoming although their range is limited to the savannah region [1,9,10,11]. Envenoming from carpet vipers leads to swelling and tissue damage at the site of bite, local and systematic bleeding, anaemia and shock. Often death results from cerebral haemorrhage, bleeding elsewhere or haemorrhagic shock [1,10,11]. The bleeding abnormality results from a prothrombin activating metalloprotease “Ecarin” and a FX activating component, an anticoagulant, platelet activator/inhibitor and haemorrhagins in the snake’s venom [1,10,11]. Non-clotting blood detected by the 20minute Whole Blood Clotting Test [20WBCT] virtually confirms carpet viper envenoming in the northern third of Africa (roughly north of the equator) and is utilized to assess adequacy of treatment [1,10,11]. Most non-carpet viper bites lead to local swelling and tissue damage. The colubrids, boomslangs and twig snake (*Thelotornis kirtlandii*), are back fanged snakes that rarely envenom but can cause severe bleeding and acute kidney injury. Neurotoxic features may result from *Naja haje*, *Naja melanoleuca* and *Dendroaspis* spp bites with deaths often resulting from respiratory muscle paralysis [12]. The risk of death from snakebites other than viper envenoming is lower [9,13,14,15,16,17], but cobra spits may lead to blindness and bites to cancerous ulcers, abortions, scarring, arthrodeses, contractures and psychological impairment leading to permanent disability and productivity loss following hospitalization and incapacitation [7,18,19,20,21]. Cessation of bleeding abnormalities and restoration of clotting following administration of effective antivenom usually occurs promptly in carpet viper envenoming. Antivenom is efficacious in decreasing the likelihood of dying and is the main treatment for snakebite envenoming [1,11,22,23]. However, its administration is associated with early adverse reactions (EAR) which rarely results in fatality.[24,25,26]. Specific interventions may be required to either prevent EAR with administration of premedication prior to antivenom or to treat it once developed following antivenom administration [25,26]. Antivenoms are formulated as either liquid agents that needs to conveyed and stored at low temperature with a life span of about three years [27,28] or as freeze dried substances that are more stable with extended shelf life. Both types of formulations have been produced for the sub-region [6,27,28]. The average cost per treatment of antivenom was reported as US$124 (range US$55–$640) although a median price of US$153 was also reported for Sub-Saharan Africa [29,30,31]. The few effective antivenoms in the sub-region generally have been scarce, locally unaffordable and inaccessible where they are most needed. Partly for these reasons antivenom utilization has drastically declined to a very small fraction of indicated need. The situation has been compounded further by the recent announcement by Sanofi-Pasteur that production and distribution of FAV Afrique, currently the most widely distributed and most dependable antivenom in the sub-region, will be discontinued by 2016. Its loss will exacerbate an already serious public health crisis and makes the management of snakebite even more challenging [32]. It is therefore extremely important within the context of other competing public health priorities to assess the health economics of antivenoms to guide policy. Before the recent publication of our work focusing on Nigeria [33], few economic evaluations of preliminary nature had been conducted on antivenoms [34,35]. Here, we evaluated the cost-effectiveness of antivenom utility in the treatment of snakebite envenomation by computing incremental cost effectiveness ratios (ICERs) of the cost per death averted and the cost per DALY averted by adapting a previously published model for Nigeria to 16 countries in WA. We performed the analysis from healthcare system perspective to provide policy makers with evidence towards broadening access to antivenoms given their importance in preventing loss of lives and limbs among poor vulnerable communities in West Africa.

## Materials and Methods

### Model Overview

**Fig 1:**
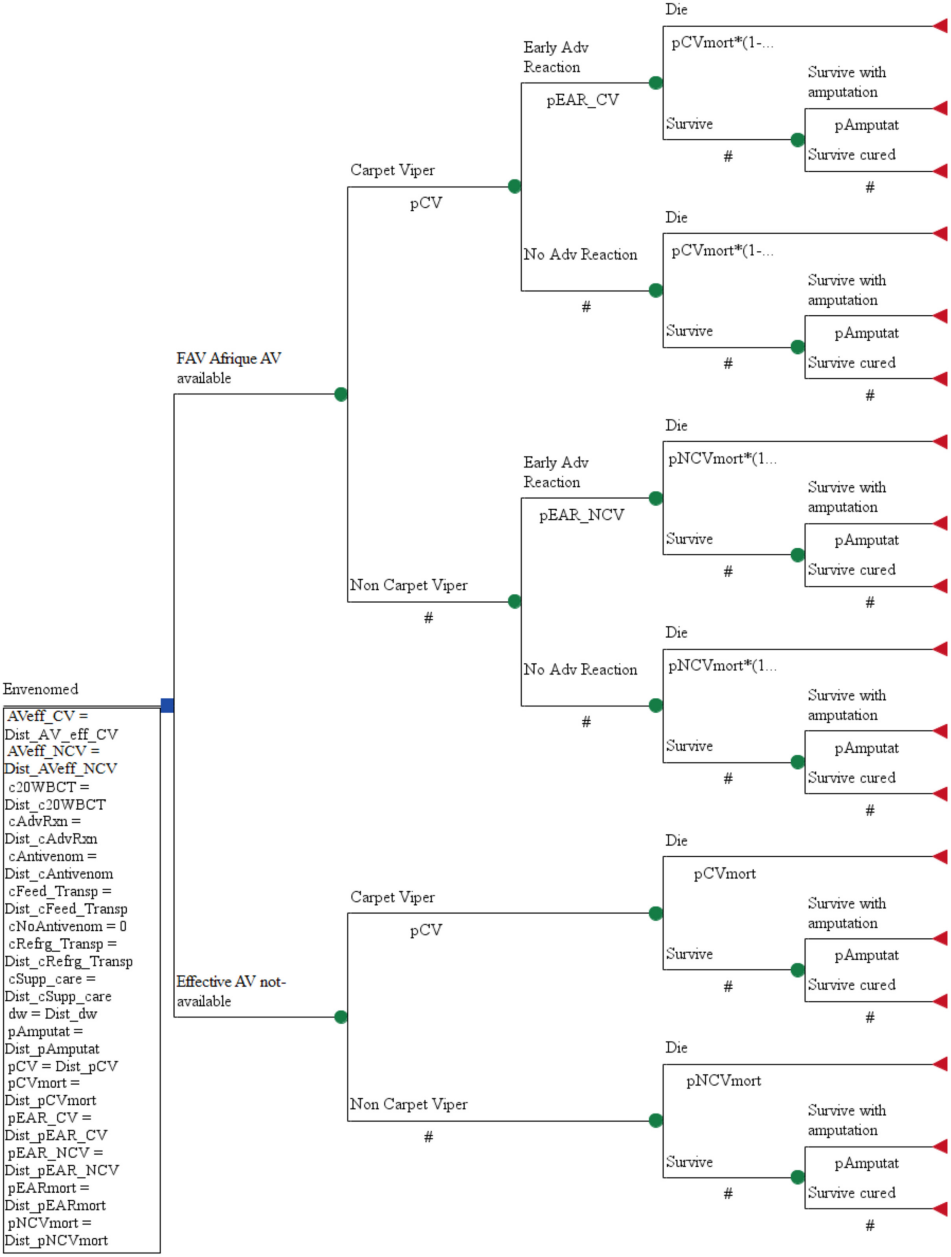
Decision tree model for managing snakebite envenoming with or without FAV Afrique antivenom in Cameroun (each of the 16 countries has a similar model with its data input). Model parameter definitions: c20WBCTest = cost of 20 minutes Whole Blood Clotting Test on 10 occassions over 7 days at diagnoses and monitoring; cAdvReaction = Cost of managing early adverse reactions; cAntivenom = Cost of Antivenom; cFeed_Transp = Cost of transporation and stay in Hospital for 7days; cRefrg_Transp = Cost of shipping and refrigeration; cNoAntivenom = Cost of management without effective antivenoms either traditional/herbal care or other alternatives; cSupp_care = Cost of supportive care. All costs are in US$. antivenomeff = Effectiveness of antivenom to prevent death; pEARmono = probability of early adverse reactionswith monospecific antivenom; pEARpoly = probability of early adverse reactionswith polyspecific antivenom; pEARmort = probability of dying following effective antivenom and early adverse reactions; pCVmort = probability of dying following carpet viper envenoming; pNCVmort = probability of dying following non-carpet viper envenoming; pCV = proportion of envenoming due to carpet viper; pDisabl = probability of disability; dw = disability weighting of consequences of snakebite envenoming; x = effect of adrenaline premedication reduction of risk of early adverse reactions.

A decision analytic model (Fig 1) was adapted to estimate health outcomes and costs associated with the availability and use of geographically appropriate and effective antivenoms for snakebite poisoning in West Africa [33]. Details of the model structure are described elsewhere [33]. Briefly, the model assessed the availability of effective antivenoms relative to no availability in the decision node. The model differentiated snakebite envenoming by carpet viper and non-carpet viper and distinction was made on the basis of the 20WBCT in the treatment arm of the model. Evidence of coagulopathy would lead to the administration of mono-specific antivenom that neutralizes carpet viper venom only, whereas absence of coagulopathy triggers the administration of a polyspecific antivenom that neutralizes venoms from several snakes, including the carpet viper. In the first chance node, the model included EARs associated with antivenom use, which are more likely to occur with polyspecific rather than the monospecific antivenom [23,27,28,36,37]. Symptoms of EAR were diverse and death could happen in about 1% of cases [24,25,26]. Survivors of snakebite may recover completely or remain with significant sequaela (e.g. amputation) that was considered in the model. Treatment outcomes were converted into DALYs on the basis of local life expectancy. Tree Age Pro Suite Healthcare 2014 software was used for analyses.

### Model Inputs

#### Antivenom effectiveness and Early Adverse Reactions (EAR) data

The likelihood of death from carpet viper and other snakebites among untreated victims was previously reported at 8.1–15.83% and 5–27.3% respectively [9,10,38,39,40,41,42] although these were varied in sensitivity analyses. When data on antivenom effectiveness was available for a given country, it was applied for that country. It ranged from 56.43% to 92% against carpet viper deaths. A meta-analysis estimate was applied for countries without data (Table 1) [38,39,40]. There hasn’t been placebo-controlled randomized controlled trials (RCT) ever conducted for antivenom assessment and the estimates of effectiveness derived from the meta-analysis that exclusively only included observational studies from Chad, Ghana and Nigeria [10,22,37,38,39] were applied in the model. Results from the meta-analysis suggested that an effective antivenom has a 75% (95% confidence interval: 55–86%) effectiveness in averting mortality from carpet viper bites [22]. Since the meta-analysis included studies wherein polyspecific antivenoms were used, these estimates of effectiveness were applied against non-carpet viper deaths except in Benin and Guinea-Conakry, where country-level effectiveness data exists for Antivipmyn antivenom against non-carpet viper (elapid) deaths, reported at 43.6% (0–80.5%) (Table 1) [42]. However, in a scenario analysis antivenoms were assumed to be ineffective (0%) against non-carpet viper envenoming. Estimates of EAR for the corresponding antivenoms were Antivipmyn 3.3% and FAV Afrique 4.3% as obtained in observational studies while it was EchiTab-G 19% and EchiTab-Plus 26% [23,36,37]. For countries where none of the antivenoms have been in use, the EAR risk median estimate of 4.3% was used in the model [36]. The EAR estimates were varied from 0% to 30% in sensitivity analyses. The risk for disability (limb amputation) among survivors was approximated to 3% from studies in sub-Saharan Africa and the tropics [7,43,44]. In base-case analysis antivenom administration had no effect on amputation, however, effectiveness was applied in sensitivity analyses. The mean ages of snakebite envenomed victims were reported for Chad 25.2 years, Niger 29 years, Nigeria 26 years and Mali 28 years [38,39,41,45,46]. So, since snakebite envenoming occurs among persons with average age in the 25–29 year age group, we determined the remaining local life expectancies in this age group for the 16 countries ranged from 37 years in Sierra-Leone to 45 years in Ghana and Senegal (Table 1) [47]. The base-case analysis on disability was restricted to extremity amputation although blindness from venom-ophthalmia and Post Traumatic Stress Disorder (PTSD) from encountering snake with respective disability weights of 0.552 and 0.105 [48] were also explored in scenario analyses. An annual discount rate of 3% was applied on the health outcomes (mortality and amputation) and the associated amputation-related disability weight used was 0.102 [48]. The model was adapted to 3 types of antivenoms used in WA: Antivipmyn in Benin and Guinea Conakry; EchiTab-G and EchiTab-Plus in Burkina Faso and Nigeria; FAV Afrique in Cameroun, Chad, Ghana and Mali. For the remaining 8 countries without evidence of effectiveness data for a particular antivenom, the meta-analytic estimates were used [22]. Where other required data item was unavailable, data from adjacent or neighboring countries were used to input a model with mixed data sources. These parameters for the respective models were obtained in the literature and imputed to the antivenom-defined models and was run for each of the 16 countries.

**Table 1:** Data estimates used in the model by country.

	(a) Mean age at bite (+remaining life expectancy [2012]), years	(b) Proportion of envenoming due to CV or vipers (%)	(c) Untreated Mortality (CV; Non-CV)	(d) Mortality post AV (CV; Non-CV)	AV effectiveness against mortality (e) = [1-RR] or alternatively = (c-d)/c (for CV; Non-CV)	Risk of Early Adverse Reactions with CV(mono) and NCV(poly[(%)	Comments	References
Model 1: Antivipmyn Antivenom								
Benin Republic	25–29 (44)	85%	15%; 9/33(27.3%)	3.11%; 4/26(15.4%)	79.3%; (NA) 43.6% (0–80.5%)	3.3%	Used NCV data from Guinea	Fayomi et al 2002 [4]; Chippaux et al 2007 [6]; Balde et al 2012 [37]; Balde et al 2013 [42];
Guinea-Conakry	25–29 (43)	83%	15%; 9/33(27.3%)	3.11%; 4/26(15.4%)	79.3%; (NA) 43.6% (0–80.5%)	3.3%	Used CV data from Benin	Fayomi et al 2002 [4]; Chippaux et al 2007 [6]; Balde et al 2012 [37]; Balde et al 2013 [42]; Adehossi et al 2011 [45];
Model 2: EchiTab-G and EchiTab-Plus Antivenom								
Nigeria	26 (41)	66%	19/120(15.83%); 5%	78/6137(1.27%)	92% (87–95%)	ET-19%; ETPlus-26%		Warrell et al 1977 [10]; Abubakar et al 2010[23]; Habib & Abubakar 2011 [38]; Pugh et al 1980 [13]
Burkina Faso	25–29 (43)	85%	12.1%; 5%	NA	92% (87–95%)	ET-19%; ETPlus-26%	Used AV effect from Nigeria; Ghana mortality	Warrell et al 1977 [10]; Abubakar et al 2010[23]; Habib & Abubakar 2011 [38]; Visser et al 2008 [40]; Pugh et al 1980 [13]
Model 3: FAV Afrique Antivenom								
Cameroon	25–29 (41)	85%	15/98(15.3%)	NA	85.2%(56.1–95%);	4.3%	Used (c) & (e) from Chad/Ghana	Chippaux et al 1999 [36]; Bregani et al 2006 [39]; Visser et al 2008 [40];
Chad	25.2 (38)	85%	15/98(15.3%)	4/60(6.67%)	56.43% (0–85.2%);	4.3%		Chippaux et al 1999 [36]; Bregani et al 2006 [39];
Ghana	25–29 (45)	85%	8/66(12.1%); -	5/278(1.8%)	85.2%(56.1–95%);	4.3%		Chippaux et al 1999 [36]; Visser et al 2008 [40];
Mali	28 (43)	85%	8.1%; 5%	1.5%	81.48% (NA)	4.3%		Chippaux et al 1999 [36]; Drame et al 2012 [41]
Other/Multiple Antivenoms								
Cote d’Ivoire	25–29 (39)	83%	12.1%;-		75%(55–86%)-	4.3%	AV efficacy from meta-analysis	Habib & Warrell 2013 [22]; Chippaux et al 1999 [36]; Visser et al 2008 [40];
Gambia	25–29 (44)	40%	14.3%		75%(55–86%)	4.3%		Enwere et al 2000 [3]; Habib & Warrell 2013 [22]; Chippaux et al 1999 [36];
Guinea-Bissau	25–29 (41)	40%	15%; 27.3%		75%(55–86%); 43.6% (0–80.5%)	4.3%		Habib & Warrell 2013 [22]; Balde et al 2013 [42]; Adehossi et al 2011 [45];
Liberia	25–29 (44)	0% (*1%)	0%(*15%);5%		75%(55–86%)	4.3%		Pugh et al 1980 [13] Habib & Warrell 2013 [22];
Niger	29 (44)	85%	15%; 5%		75%(55–86%)	4.3%		Habib & Warrell 2013 [22]; Chippaux et al 1999 [36]; Fayomi et al 2002 [4]; Adehossi et al 2011 [45];
Senegal	25–29 (45)	40%	15%;5%		75%(55–86%)	4.3%		Trape et al 2002 [5];Habib & Warrell 2013 [22]; Chippaux et al 1999 [36]; Adehossi et al 2011 [45];
Sierra-Leone	25–29 (37)	0% (*1%)	0% (*15%);5%		75%(55–86%)	4.3%		Pugh et al 1980 [13] Habib & Warrell 2013 [22];
Togo	25–29 (43)	85%	12.1%; 5%		75%(55–86%)—	4.3%		Habib & Warrell 2013 [22]; Visser et al 2008 [40];

CV—carpet viper; RR—Relative Risk

#### Cost data

The cost of the full antivenom treatment regimen was modeled as US$153 [29,30,31]. The cost of care, ten 20WBCT measurements in 7 days, transportation to-and-from hospital and feeding for 7 days, shipping and freezing of antivenom, management of EAR, supportive care (such as pain relief, blood transfusion, medications, fluid replacement and wound management) were obtained from series of envenomed patients admitted to Kaltungo General Hospital in Nigeria Table 2 [33]. All costs were expressed in US$. Given snakebite is a short term condition and costs occur during a brief period of time (2 to 10 days), costs were not discounted to adjust for time elapsed between expenditure and outcome during Incremental Cost Effectiveness Ratio (ICER) calculations [49,50]. As a conservative analysis the alternative of no antivenom therapy carried a cost of zero as no treatment, care, test and EAR management was provided. However, in a scenario analysis cost of $65.63 was added/incurred for the alternative of no antivenom therapy, comprising of supportive care $18.75, feeding and transportation $43.75, and for 20WBCT $3.125. The ICER was computed by dividing total cost by the difference in DALYs (e.g., cost/DALY averted) [49,50].

**Table 2:** General assumptions used in Monte Carlo simulations.

Assumption	Base Case Value (BCV)	Range of BCVs	Distribution	Reference
Proportion of envenoming due to CV/ Non-Elapids (%)	Varies by country	1–85%	Beta	1,5,6,7,36
Mortality due to untreated CV envenoming (%)	Varies by country	8.1–15.83%	Beta	3,4,10,39,40,41,45
Mortality due to untreated Non-CV/Elapid envenoming (%)	Varies by country	5–27.3%	Beta	13,14,42
AV effectiveness against CV mortality (%)	Varies by country	56.43–92%	Beta	4,6,10,22,38,39,40,41
AV effectiveness against Non-CV/Elapid mortality (%)	Varies by country	43.6–92%	Beta	10,38,42
Risk of AV EAR for CV envenoming (%)	Varies by country	3.3–19%	Beta	23,36,37
Risk of AV EAR for Non-CV envenoming (%)	Varies by country	3.3–26%	Beta	23,36,37
Risk of AV EAR mortality (%)	1%	Same for each country	Beta	24
Risk of amputation following envenoming (%)	3%	Same for each country	Beta	7,43,44
Disability weight for amputation	0.102	Same for each country	Beta	48
Cost of antivenom (US$)	$153	Same for each country	Normal	29,30,31
Cost of 20min Whole Blood Clotting Test	$3.125	Same for each country	Normal	33
Cost of managing Early Adverse Reactions	$1.875	Same for each country	Normal	33
Cost of supportive care	$18.75	Same for each country	Normal	33
Cost of feeding and transportation	$43.75	Same for each country	Normal	33
Cost of refrigeration and transportation	$18.75	Same for each country	Normal	33
Cost of no antivenom (US$)	$0	Same for each country	Not varied	Our assumption
DALYs averted per death averted (3% discounted)	Varies by country	22.17–24.52	Not varied	Our calculations
DALYs averted per amputation averted (3% discounted)	Varies by country	2.26–2.50	Not varied	Our calculations

#### Sensitivity analysis

One-way sensitivity analysis was performed for select input variables to test robustness and determine the most important variables influencing cost-effectiveness of an antivenom programme. Each base-case model input was varied independently according to the upper and lower limits obtained from literature and according to low and high value scenarios.

In addition, Probablistic Sensitivity Analyses (PSA) were performed using Monte Carlo simulation by running 10,000 iterations of the model while randomly selecting the values for 16 key model inputs from a probability distribution that was defined for each of the parameters (Table 2). This process enabled us to estimate the 95% confidence interval around the base case ICER estimates for each of the 16 countries included in the analysis, and it also allowed us to estimate the probability that antivenom therapy is cost-effective by calculating the proportion of 10,000 iterations that resulted in ICERs that fell below commonly accepted cost-effectiveness thresholds of one time per capita Gross Domestic Product (GDP) for each of the 16 countries [51,52].

The study reporting was done consistent to standard guidelines for cost effectiveness analyses and the Consolidated Health Economic Evaluation Reporting Standards (CHEERS) statement [49,50,52,53,54].

## Results

The cost/death averted for the 16 countries of interest varied. It was as low as $1,997 in Guinea Bissau to as high as $6,205 in Liberia and Sierra Leone. The cost/DALY averted ranged from a low of $83 (95% Confidence Interval: $36-$240) for Benin Republic to a high of $281 ($159–457) for Sierra-Leone. In all cases, the base-case cost/DALY averted estimate fell below the commonly accepted threshold of one time per capita GDP, suggesting that AV is highly cost-effective for the treatment of snakebite in all 16 WA countries [51,52].

**Fig 2:**
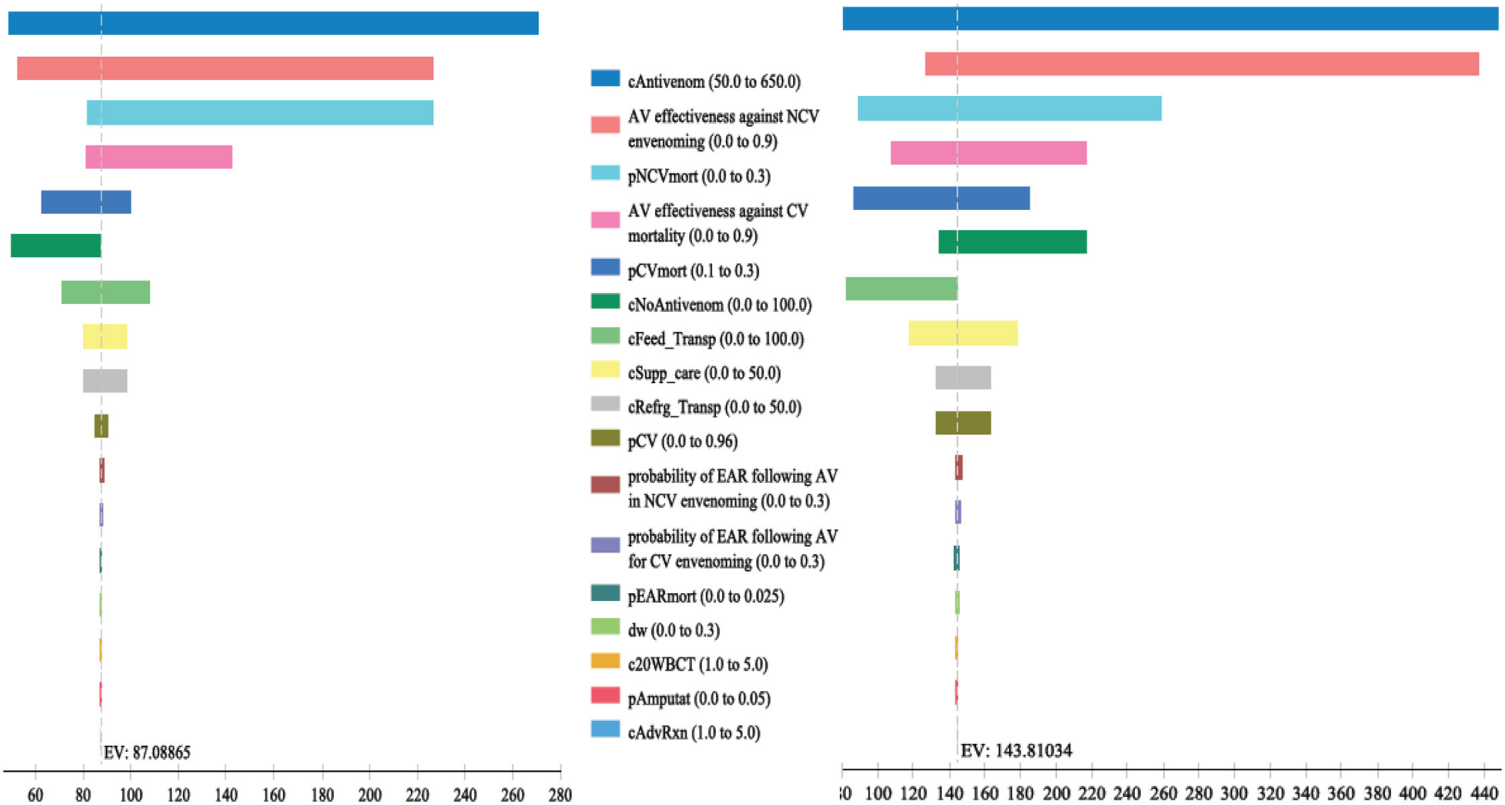
Tornado diagrams assessing the impact of changes in envenoming/antivenom and cost parameters on the incremental cost-effectiveness ratio (ICER) per DALY for antivenom use in Guinea Bissau (L) and Senegal (R). Diagram parameter definitions: c20WBCTest = cost of 20 minutes Whole Blood Clotting Test on 10 occassions over 7 days at diagnoses and monitoring; cAntivenom = Cost of Antivenom; cFeed_Transp = Cost of transporation and stay in Hospital for 7days; cRefrg_Transp = Cost of shipping and refrigeration; cNoAntivenom = Cost of management without effective antivenoms either traditional/herbal care or other alternatives; cSupp_care = Cost of supportive care. All costs are in US$. Antivenomeff = Effectiveness of antivenom to prevent death; pEARmono = probability of early adverse reactions with monospecific antivenom; pEARpoly = probability of early adverse reactionswith polyspecific antivenom; pEARmort = probability of dying following effective antivenom and early adverse reactions; pCVmort = probability of dying following carpet viper envenoming; pNCVmort = probability of dying following non-carpet viper envenoming; pCV = proportion of envenoming due to carpet viper; pDisabl = probability of disability.

The findings from the analyses were also consistent to variations of inputs in 1-way sensitivity and scenario analyses as depicted (Table 3 and Fig 2). The individual countries’ model results were most sensitive to effectiveness of antivenom in decreasing mortality, natural (unattended) mortality, costs of antivenoms and types of snake causing envenoming (Fig 2). Results were not sensitive to antivenom associated EAR or the cost of managing it. Varying the cost of antivenom from $125 to two times for victims who may require two doses, i.e. $306, still yielded ICER estimates that remain cost-effective. The ICERs rose when the frequency of snakebite envenomation due to saw-scaled viper was reduced to 0% except in Benin and Guinea Conakry where Antivipmyn antivenom is used and is effective even against elapids (Table 3) [42].

**Table 3:** Results from model outputs by country and scenarios.

Country and GDP/Capita ($) [49]	Increm Cost Effect Ratio[ICER]/DALY ($) (95% Conf. Interval)	Cost/Death Averted ($)	Probability Antivenom is cost-effective (%)	ICER if Antivenom Cost = $125	ICER if Antivenom Cost = $306	ICER if proportion of Carpet Viper = 0% ($)	ICER if Av Effect for Non Carpet Viper = 0% ($)	ICER if the ‘No Antivenom’ arm paid for Basic costs of $65.63*
Benin (751)	82.63 (36.41–240.09)	1997.91	99.99	72.87	135.96	81.75	97.26	59.75
B/Faso (652)	99.44 (40.39–377.40)	2384.81	99.61	87.98	164.05	226.53	107.18	71.94
Cameroun (1220)	86.97 (38.47–240.43)	2030.05	100.00	76.70	143.11	238.39	92.01	62.89
Chad (1035)	136.94 (51.33–704.75)	3070.80	99.13	120.77	225.34	376.61	144.89	99.03
Cote d’Ivoire (1366)	128.24 (51.20–461.64)	2916.02	99.97	113.09	211.04	278.37	139.16	92.73
Gambia (509)	150.08 (72.18–305.49)	3628.88	99.99	132.25	247.47	261.77	229.59	108.30
Ghana (1646)	103.61 (42.04–372.87)	2532.73	99.99	91.38	170.50	227.63	111.21	74.93
Guinea Bissau (576)	87.09 (44.96–171.55)	2032.72	100.00	76.75	143.60	84.76	226.64	62.85
Guinea Conakry (493)	83.54 (36.59–236.35)	1997.41	99.98	73.67	137.49	82.68	100.72	60.40
Liberia (414)	256.61 (147.67–417.68)	6204.95	97.28	226.00	423.92	261.77	13,964.26	184.85
Mali (696)	160.48 (82.21–306.83)	3836.74	100.00	141.52	264.06	243.47	178.09	116.04
Niger (385)	97.23 (39.84–328.02)	2351.06	98.64	85.75	159.99	261.77	102.98	70.31
Nigeria (2742)	92.56 (40.27–242.63)	2160.33	100.00	81.61	152.35	232.04	107.96	66.91
Senegal (1023)	143.81 (67.34–317.76)	3515.25	100.00	126.73	237.14	258.95	216.41	103.78
Sierra Leone (590)	280.77 (158.51–456.68)	6204.95	99.86	247.27	463.83	286.42	15,278.99	202.25
Togo (589)	120.42 (47.62–455.04)	2878.98	98.86	106.19	198.14	264.75	129.25	87.08

*Scenario of Basic costs in the No antivenom arm = Cost of supportive care ($18.75) + Cost of feeding and transportation ($43.75) + Cost of 20min Whole Blood Clotting Test ($3.125) = $65.63

AV—antivenom; ICER—Incremental Cost Effectiveness Ratio;

Moreover, the ICER ranged from $97.26 in Benin to high levels of $13,964.26 in Liberia and $15,278.99 in Sierra Leone even in the worst case scenario where (poly-specific) antivenoms have nil effectiveness (0%) against bites from snakes other than carpet viper. These estimates fall outside the cost-effectiveness thresholds in Liberia and Sierra Leone largely because non-carpet viper accounts for 99% of SBE. Applying a modest reduction of 40% on the probability of EAR with the use of adrenaline premedication [25,26,33] gave a cost per DALY averted slightly lower than base-case ICERs. Similarly, the ICERs were only very slightly altered even when more serious or more frequent disabilities were substituted in the model. This was demonstrated with venom-induced-blindness (0.01%) or Post-Traumatic-Stress-Disorder (20%) with disability-weights of 0.552 and 0.105 respectively [18,21,48].

**Fig 3:**
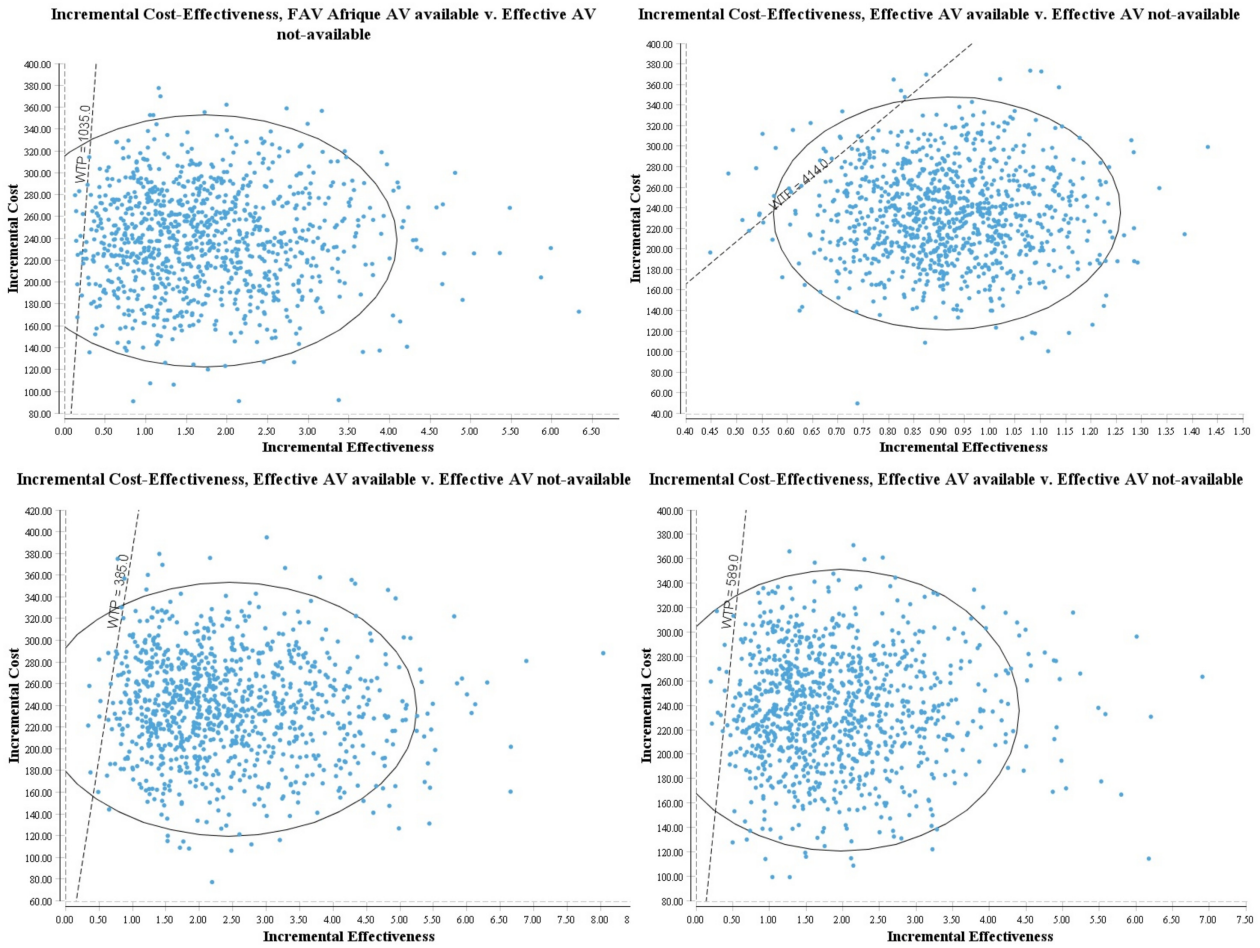
Monte Carlo Simulation showing the probability antivenom is cost-effective in majority of iterations (out of 10,000) for 4 countries with respective Willingness To Pay (GDP/capita) and probabilities: top panel Chad (WTP $1035; prob-99.13%) [L] and Liberia (WTP $414; prob-97.28%) [R] and bottom panel Niger (WTP $385; prob-98.64%) [L] and Togo (WTP $589; prob-98.86%) [R].

Furthermore, our PSA confirms the model findings remain consistent to concurrent variation of all model inputs, as the ICERs with their respective 95% confidence limits are far less than the cost-effectiveness thresholds (Table 3). It showed that in majority of simulations (97.3% in Liberia to 100% in Cameroun, Guinea Bissau, Mali, Nigeria and Senegal (Fig 3)) our model results yielded an ICER that fell below the threshold of one time per capita GDP, thus, indicating a high degree of confidence in our results [51,52].

## Discussion

Economic modeling is very useful in determining the best ways to utilize resources to optimally manage medical conditions where there are competing priorities and limited resources [49]. This is the first extensive assessment of the cost-effectiveness of expanding antivenom access in the 16 countries in West Africa. We find that the cost/death averted for the 16 countries of interest ranged from $1,997 in Guinea Bissau to $6,205 for Liberia and Sierra Leone. The cost/DALY averted ranged from $83 (95% Confidence Interval: $36-$240) for Benin Republic to $281 ($159–457) for Sierra-Leone. The ICER point estimate is <$100/DALY averted in 7 countries, $100-$200/DALY averted in 7 countries and <$300/DALY averted in 2 countries. The results show that snakebite antivenoms are highly cost-effective in West Africa, as our findings are far less than the one time per capita GDP threshold [51,52]. While it will be worthwhile to repeat the analysis for similar geographic and socioeconomic settings, most of the model inputs such as the antivenom efficacy and cost would largely be similar across many African countries with similar GDPs where snakebite envenoming occurs. An exception may be the prevalence of carpet viper (*Echis ocellatus*) envenoming, a snake that is confined to West Africa extending eastwards only as far as Chad and Central Africa Republic. However, even in areas without carpet viper bites (0%) our results demonstrate that antivenoms remain highly cost-effective.

The combination of effective and relatively inexpensive antivenoms and the utilization of a cheap, dependable and simple point-of-care test have been instrumental to our results. Antivenom effectiveness is loosely inversely related to the ICER per DALY saved (Fig 2) [33]. With discontinuation of production of geographically appropriate effective antivenoms and marketing of inappropriate ineffective products the cost per DALY saved will substantially soar [32,39,40]. The 20WBCT is discriminatory and will be useful following envenoming from other snakes in the rest of Africa that result in coagulopathy e.g., other species of carpet viper (*Echis leucogaster*, *Echis pyramidum*, *Echis jogeri*, *Echis coloratus*) and boomslang. In the context of differing circumstances where multiple types of snakes with varying manifestations of envenoming or lack of reliable cheap differentiating test, antivenoms may not be as cost-effective. Nevertheless, where patients come along with the dead snakes, decision on antivenom choice is feasible [23] and in such cases the differentiating test becomes not so useful. In the face of competing health needs and constrained-resources, it would be helpful to contextualize our findings in the light of other healthcare initiatives. The cost-effectiveness of Human Immunodeficiency Virus treatment used in similar resource-constrained settings as first-line, second-line or for protecting negative partners among discordant partners ranged from US$530 to US$1037 per year of life gained [33,55,56,57]. However, these estimates are still higher than the highest ICERs obtained for two countries in this analysis, i.e., Liberia and Sierra-Leone with $257/DALY averted and $281/DALY averted respectively. The antivenom cost effectiveness is comparable to what obtains in other healthcare programmes. For example, the cost/DALY averted obtained in this study ranged from $100 to $200 for 7 countries (see Table 3) and is comparable to the cost effectiveness of rotavirus vaccines in other developing countries in Africa and Asia [33,58,59]. Similarly, the cost/DALY averted in the remaining 7 countries was <$100/DALY averted and is similar to what obtains for preventing Human Papilloma Virus and pneumococcal infections with vaccines in West Africa [33,60,61].

We estimated a cost/DALY averted ranging from $73 in Benin to $247 in Sierra Leone if the cost input of antivenom is reduced to $125 per dose as obtained in Mali [30]. Doubling the cost of antivenom for patients requiring more than a dose still yielded ICERs that remain cost effective ranging from $136/DALY averted in Benin to $464/DALY averted in Sierra Leone.

In 8 of the 16 countries with neither indigenous antivenom effectiveness data nor data from adjacent countries, we used efficacy derived from a meta-analysis with data inputs from other countries in the sub-region [22, 38].

The study has a number of limitations. First, the effectiveness of antivenom was derived from observational studies rather than RCTs. Definitive placebo controlled trials of antivenom are considered unethical and RCTs are unlikely to be conducted in the absence of a suitable comparator to antivenom. However, to reduce bias, improve data quality and validity of estimates, three investigators independently searched both English and French literature and extracted data using a checklist for consistency. Secondly, we applied the estimated protection conferred by antivenoms against carpet viper envenoming to the antivenoms used for other than carpet viper envenoming (except in Benin and Guinea Conakry with specific estimates), though this assumption was subsequently dropped in a scenario analysis where antivenoms were assumed to be ineffective (0%) against non-carpet viper envenoming. Thirdly, the analysis mainly considered amputation as the major disability to the exclusion of other anecdotal complications [7, 20, 43, 44]. Fourthly, in our model, antivenoms only conferred protection against death—being a more objective and valid outcome. Fifthly, other benefits of antivenom (e.g. speedier recovery) were not included in the model. Sixth, we used separate EAR risk inputs for EchiTab-G and EchiTab-Plus respectively [23] but could not discern their respective efficacy against mortality so the combined estimate of the two was used in Burkina Faso and Nigeria. Lastly, we did not include the costs incurred for logistics in conveying and preserving antivenoms as we assumed facilities for existing health programmes will be utilized.

### Conclusion

The findings from the cost effectiveness analysis demonstrate that providing and broadening antivenom access throughout areas at risk in rural West Africa should be prioritized given the considerable reduction in deaths and disabilities that could be derived at a relatively small cost.

## References

[1] Warrell DA and Arnett C. The importance of bites by the saw-scaled or carpet viper (Echis carinatus). Epidemiological studies in Nigeria and a review of world literature Acta Tropica 1976; 33, 307–341. 14490

[2] Visser L.E., Kyei-Faried S., Belcher D.W., et al Protocol and monitoring to improve snake bite outcomes in rural Ghana. Trans. R. Soc. Trop. Med. Hyg. 2004; 98: 278–283. 15109550 10.1016/S0035-9203(03)00065-8

[3] Enwere GC, Obu HA, Jobarteh A. Snake bites in children in The Gambia. Ann Trop Paediatr. 2000 6;20(2):121–4. (14.3%) 10945062 10.1080/02724936.2000.11748120

[4] Fayomi B, Massougbodji A, Chobli M. Epidemiological data on snake bite cases reported in Benin from 1994 to 2000. Bull Soc Pathol Exot. 2002 8;95(3):178–80. (15% lethality) 12404865

[5] Trape JF, Pison G, Guyavarch E, Mane Y. Mortality from snake bites, wild and domestic animal bites and arthropod stings in the savannah zone of eastern Senegal. Bull Soc Pathol Exot. 2002 8;95(3):154–6 (40% echis) 12404858

[6] Chippaux JP, Massougbodji A, Stock RP, Alagon A; Investigators of African Antivipmyn in Benin. Clinical trial of an F(ab')2 polyvalent equine antivenom for African snake bites in Benin. Am J Trop Med Hyg. 2007 9;77(3):538–46. (post AV mortality 3.11%) 17827375

[7] Chippaux J-P. Estimate of the burden of snakebites in sub-Saharan Africa: A meta-analytic approach. Toxicon 2011. 10.1016/j.toxicon.2010.12.022 21223975

[8] Kasturiratne A, Wickremasinghe AR, de Silva NR, et al The global burden of snakebite: a literature analysis and modelling based on regional estimates of envenoming and deaths. PLoS Medicine 2008; 5 (11): 1591–1604 10.1371/journal.pmed.0050218 PMC2577696 18986210

[9] Pugh RNH and Theakston RDG. Incidence and mortality of snake bite in savannah Nigeria. Lancet 1980; 2, 1181–3. 6107780 10.1016/s0140-6736(80)92608-2

[10] Warrell DA, Davidson NMcD, Greenwood BM et al Poisoning by bites of the saw-scaled or carpet viper (Echis carinatus) in Nigeria. Quart. J. Med (ns) 1977; 46, 33–62. 866568

[11] Meyer WP, Habib AG. Onayade AA et al First clinical experience with a new ovine Fab Echis ocellatus snake bites antivenom in Nigeria. Randomized comparative trial with institute Pasteur Serum (IPSer) Africa antivenom Amer, J. Trop. Med & Hyg. 1997; 56(3), 292–300. 10.4269/ajtmh.1997.56.291 9129531

[12] Warrell DA, Barnes HJ and Pirburn MF, Neurotoxic effects of bites by the Egyptian cobra (Naja haje) in Nigeria 1974 Trans. Roy. Soc. Trop. Med & Hyg. 1974; 70, 78–80. 10.1016/0035-9203(76)90012-2 1265823

[13] Pugh RNH, Theakston RDG, Reid HA and Bhar IS. Malumfashi endemic diseases research project XIII. Epidemiology of human encounters with the spitting cobra (Naja nigricollis) in the Malumfashi area of northern Nigeria. Ann. Trop. Med. & Parasitol. 1980; 74,523–30. 7469568

[14] Muguti GI, Maramba A, Washaya CT. Snakebites in Zimbabwe: a clinical study with emphasis on the need for antivenom. Central African Journal of Medicine 1994; 40(4): 83–8 7954715

[15] Warrell DA, Ormerod LD and Davidson NMcD. Bites by puff adder (Bitis arietans) in Nigeria and value of antivenom. Brit. Med. Journ. 1975; 4, 697–700. 10.1136/bmj.4.5998.697 PMC1675831 1203728

[16] Warrell DA, Ormerod LD and Davidson NMcD. Bites by night adder (Causus maculatus) and burrowing vipers (Genus Atractaspis) in Nigeria 1976 Am Jour. Trop. Med. & Hyg. 1976; 25, 517–524. 945703 10.4269/ajtmh.1976.25.517

[17] Warrell DA, Greenwood BM, Davidson NMcD et al Necrosis, haemorrhage and complement depletion following bites by the spitting cobra (Naja nigricollis) 1976 Quart. J Med (ns) 1976; 45, 1–22. 943796

[18] Warrell DA and Ormerod LD. Snake venom ophthalmia and blindness caused by the spitting cobra (Naja nigricollis) in Nigeria 1976 Amer. J. Trop. Med & Hyg. 1976c; 25, 525–529. 1084700 10.4269/ajtmh.1976.25.525

[19] Mustapha SK, Mubi BM, Askira BH. Bilateral blindness following snakebite. Trop Doct. 2010 4;40(2):117–8. 10.1258/td.2009.090429 20305112

[20] Habib AG, Abubakar SB, Abubakar IS, Larnyang S, Durfa N, Nasidi A, Yusuf PO, Garnvwa J, Theakston RDG, Salako L, Warrell DA. Envenoming following carpet viper (Echis ocellatus) bite during pregnancy: timely use of effective antivenom improves materno-fetal outcomes. Tropical Medicine and International Health 2008; 13(9): 1172–5 10.1111/j.1365-3156.2008.02122.x 18631310 PMC2857546

[21] Williams SS, Wijesinghe CA, Jayamanne SF, Buckley NA, Dawson AH, Lalloo DG, de Silva HJ. Delayed Psychological Morbidity Associated with Snakebite Envenoming. PLoS Neg Trop Dis 2011; 5(8): e1255. 10.1371/journal.pntd.0001255 PMC3149015 21829741

[22] Habib AG and Warrell DA. Antivenom therapy of carpet viper (Echis ocellatus) envenoming: effectiveness and strategies for delivery in West Africa. Toxicon 2013;69:82–89 10.1016/j.toxicon.2013.01.002 23339853

[23] Abubakar IS, Abubakar SB, Habib AG, Nasidi A, Durfa N, Yusuf PO, Larnyang S, Garnvwa J, Sokomba E, Salako L, Theakston RD, Juszczak E, Alder N, Warrell DA; Nigeria-UK EchiTab Study Group. 2010 Randomised controlled double-blind non-inferiority trial of two antivenoms for saw-scaled or carpet viper (Echis ocellatus) envenoming in Nigeria. PLoS Negl Trop Dis. 4(7):e767 10.1371/journal.pntd.0000767 20668549 PMC2910709

[24] Williams DJ, Jensen SD, Nimorakiotakis B, Müller R, Winkel KD. Antivenom use, premedication and early adverse reactions in the management of snake bites in rural Papua New Guinea. Toxicon. 2007 5;49(6):780–92. Epub 2006 Dec 2. 17210167 10.1016/j.toxicon.2006.11.026

[25] Habib AG. Effect of pre-medication on early adverse reactions following antivenom use in snakebite: a systematic review and meta-analysis. Drug Saf. 2011 10 1;34(10):869–80. 10.2165/11592050-000000000-00000 21879781

[26] de Silva HA, Pathmeswaran A, Ranasinha CD, Jayamanne S, Samarakoon snakebite, Hittharage A, Kalupahana R, Ratnatilaka GA, Uluwatthage W, Aronson JK, Armitage JM, Lalloo DG, de Silva HJ. Low-dose adrenaline, promethazine, and hydrocortisone in the prevention of acute adverse reactions to antivenom following snakebite: a randomised, double-blind, placebo-controlled trial. PLoS Med. 2011 5;8(5):e1000435 10.1371/journal.pmed.1000435 21572992 PMC3091849

[27] EchiTab study group on monospecific antivenom; http://www.lstmliverpool.ac.uk/research/departments/parasitology/venom-unit/echitab-study-group/ (accessed on 8 Aug 2015)

[28] EchiTab IgG Plus ICP Polyspecific antivenom product description. http://www.echitabplusicp.org/product/description.html (accessed on 8 Aug 2015)

[29] Brown NI. Consequences of neglect: analysis of the sub-Saharan African snake antivenom market and the global context. PLoS Negl Trop Dis. 2012;6:e1670 10.1371/journal.pntd.0001670 22679521 PMC3367979

[30] Dramé BS, Dabo M, Diani N, Cissé B. Assessment of the availability and use of antivenom in the district of Bamako,Mali, West Africa. Bull Soc Pathol Exot. 2012 8;105(3):179–83. 22707256 10.1007/s13149-012-0239-8

[31] Management Sciences for Health. International Drug Price Indicator Guide. 2012 edition. Cambridge, MA, USA: Management Sciences for Health; 2013.

[32] Chippaux JP, Habib AG. Antivenom shortage is not circumstantial but structural. Trans R Soc Trop Med Hyg. 2015 12;109(12):747–8. 10.1093/trstmh/trv088 26626337

[33] Habib AG, Lamorde M, Dalhat MM, Habib ZG, Kuznik A. Cost-effectiveness of Antivenoms for Snakebite Envenoming in Nigeria. PLoS Neglected Tropical Diseases 2015; 9(1):e3381 10.1371/journal.pntd.0003381 25569252 PMC4287484

[34] Morais V, Massaldi H. Economic evaluation of snake antivenom production in the public system. Journal of Venomous Animals and Toxins including Tropical Diseases 2006; 3: 497–511

[35] Brown N and Landon J. Antivenom: The most cost-effective treatment in the world? Toxicon 2010; 55: 1405–7 10.1016/j.toxicon.2010.02.012 20171241

[36] Chippaux JP, Lang J, Amadi-Eddine S, Fagot P, Le Mener V. Short report: treatment of snake envenomations by a new polyvalent antivenom composed of highly purified F(ab)2: results of a clinical trial in northern Cameroon. Am J Trop Med Hyg. 1999 12;61(6):1017–8 10674688 10.4269/ajtmh.1999.61.1017

[37] Baldé MC, Chippaux JP, Boiro MY, Stock R, Massougbodji A. Clinical study of tolerance and effectiveness of a F(ab')(2) polyvalent antienom for African snake bites in Kindia, Guinea. Bull Soc Pathol Exot. 2012 8;105(3):157–61. 10.1007/s13149-012-0223-3 Epub 2012 Feb 22 22359185

[38] Habib AG, Abubakar SB 2011 Factors affecting snakebite mortality in north-eastern Nigeria. International Health. 3: 50–55 10.1016/j.inhe.2010.08.001 24038050

[39] Bregani ER, Cantoni F, Tantardini F 2006 Snake bites in South Chad. Comparison between three different polyvalent anti-snake venom immunotherapies. Italian Journal of Tropical Medicine 11: (1–2); 25–28

[40] Visser LE, Kyei-Faried S, Belcher DW, Geelhoed DW, van Leeuwen JS, van Roosmalen J, 2008 Failure of a new antivenom to treat Echis ocellatus snake bite in rural Ghana: the importance of quality surveillance. Trans R Soc Trop Med Hyg 102(5):445–50. 10.1016/j.trstmh.2007.11.006 18190937

[41] Dramé BS, Diarra A, Diani N, Dabo A. Epidemiological, clinical and therapeutics aspects of snakebites in the Gabriel-Touré and Kati national hospitals of Mali: a ten-year retrospective study. Bull Soc Pathol Exot. 2012 8;105(3):184–8. Epub 2012 Jun 16 10.1007/s13149-012-0240-2 22707257

[42] Baldé MC, Chippaux JP, Boiro MY, Stock RP, Massougbodji A. Use of antivenoms for the treatment of envenomation by Elapidae snakes in Guinea, Sub-Saharan Africa. J Venom Anim Toxins Incl Trop Dis. 2013 3 28;19(1):6 10.1186/1678-9199-19-6 23849079 PMC3707107

[43] Laohawiriyakamol S, Sangkhathat S, Chiengkriwate P, Patrapinyokul S. Surgery in management of snake envenomation in children. World J Pediatr. 2011 11;7(4):361–4. Epub 2011 Aug 27. 10.1007/s12519-011-0282-8 21877258

[44] Yates VM, Lebas E, Orpiay R, Bale BJ. Management of snakebites by the staff of a rural clinic: the impact of providing free antivenom in a nurse-led clinic in Meserani, Tanzania. Ann Trop Med Parasitol. 2010 7;104(5):439–48. 10.1179/136485910X12743554760306 20819312

[45] Adehossi E, Sani R, Boukari-Bawa M, Niaouro S, Gbaguidi F, Abdou I, Parola P. Snake bites in the emergency unit of Niamey National Hospital, Niger. Bull Soc Pathol Exot. 2011 12;104(5):357–60. Epub 2011 Aug 1. 21809217 10.1007/s13149-011-0152-6

[46] Habib AG, Kuznik A, Hamza M, Abdullahi MI, Chedi BA, Chippaux JP, Warrell DA. Snakebite is Under Appreciated: Appraisal of Burden from West Africa. PLoS Negl Trop Dis. 2015 9 23;9(9):e0004088 10.1371/journal.pntd.0004088 26398046 PMC4580425

[47] World Health Organization. Mortality and life expectancy data Nigeria. Global Health Observatory, WHO, 2012. http://apps.who.int/gho/athena/data/download.xsl?format=xml&target=GHO/LIFE_0000000029,LIFE_0000000030,LIFE_0000000031,LIFE_0000000032,LIFE_0000000033,LIFE_0000000034,LIFE_0000000035&profile=excel&filter=COUNTRY:XYZ

[48] World Health Organization. Global Burden of Disease 2004 Update: Disability weights for diseases and conditions, WHO

[49] Hunink MG and Glasziou P 2001 Decision making in health and medicine. Cambridge University Press, pp 1–388

[50] Fox-Rushby JA and Hanson K. Calculating and presenting disability adjusted life years (DALYs) in cost-effectiveness analysis. Health Policy and Planning 2001; 16(3): 326–331. 11527874 10.1093/heapol/16.3.326

[51] World Bank 2012 GDP per capita. Washington, DC: World Bank—http://data.worldbank.org/indicator/NY.GDP.PCAP.CD (accessed 13 June 2015)

[52] World Health Organization. Cost effectiveness thresholds. Geneva: World Health Organization, 2011.

[53] Siegel JE, Weinstein MC, Russell LB, Gold MR. Recommendations for reporting cost-effectiveness analyses. Panel on Cost-Effectiveness in Health and Medicine. JAMA. 1996 10 23–30;276(16):1339–41. 8861994 10.1001/jama.276.16.1339

[54] Husereau D, Drummond M, Petrou S, Carswell C, Moher D, Greenberg D, Augustovski F, Briggs AH, Mauskopf J, Loder E. Consolidated Health Economic Evaluation Reporting Standards (CHEERS) statement. Eur J Health Econ. 2013;14(3):367–372. 10.1007/s10198-013-0471-6 23526140

[55] Goldie SJ, Yazdanpanah Y, Losina E, Weinstein MC, Anglaret X, Walensky RP, et al Cost effectiveness of HIV treatment in resource-poor settings—the case of Cote d’Ivoire. N Engl J Med 2006; 355: 1141–1153 16971720 10.1056/NEJMsa060247

[56] Long L, Fox M, Sanne I, Rosen S. The high cost of second-line antiretroviral therapy for HIV/AIDS in South Africa. AIDS 2010; 24: 915–919 10.1097/QAD.0b013e3283360976 20042849

[57] Walensky RP, Ross EL, Kumarasamy N, Wood R, Noubary F, Paltiel D, et al Cost effectiveness of HIV treatment as prevention in serodiscordant couples. N Engl J Med 2013; 369: 1715–1725 10.1056/NEJMsa1214720 24171517 PMC3913536

[58] van Hoek AJ, Ngama M, Ismail A, Chuma J, Cheburet S, Mutonga D, Kamau T, Nokes DJ. A cost effectiveness and capacity analysis for the introduction of universal rotavirus vaccination in Kenya: comparison between Rotarix and RotaTeq vaccines. PLoS One. 2012;7(10):e47511 Epub 2012 Oct 24. 10.1371/journal.pone.0047511 23115650 PMC3480384

[59] Patel HD, Roberts ET, Constela DO. Cost-effectiveness of a new rotavirus vaccination program in Pakistan: a decision tree model. Vaccine 2013; 31: 6072–8. 10.1016/j.vaccine.2013.10.022 24176497 PMC3865920

[60] Goldie SJ, O’Shea M, Campos NG, Dias M, Sweet S, Kim SY. Health and economic outcomes of HPV 16, 18 vaccination in 72 GAVI eligible countries. Vaccine 2008; 26(32): 4080–93. 10.1016/j.vaccine.2008.04.053 18550229

[61] Sinha A, Levine O, Knoll MD, Muhib F, Lieu TA. Cost effectiveness of pneumococcal conjugate vaccination in the prevention of child mortality: an international economic analysis. Lancet 2007; 369:389–96 17276779 10.1016/S0140-6736(07)60195-0

